# The Use of Stromal Vascular Fraction in Long Bone Defect Healing in Sheep

**DOI:** 10.3390/ani13182871

**Published:** 2023-09-09

**Authors:** Elena I. Pappa, Mariana S. Barbagianni, Stefanos G. Georgiou, Labrini V. Athanasiou, Dimitra Psalla, Dionysios Vekios, Eleni I. Katsarou, Natalia G. C. Vasileiou, Pagona G. Gouletsou, Apostolos D. Galatos, Nikitas N. Prassinos, Dimitris A. Gougoulis, Marianna Angelidou, Vicky Tsioli, George C. Fthenakis, Aikaterini I. Sideri

**Affiliations:** 1 Faculty of Veterinary Science, University of Thessaly, 43100 Karditsa, Greece; 2 School of Veterinary Medicine, Aristotle University of Thessaloniki, 54124 Thessaloniki, Greece; 3 School of Medicine, Aristotle University of Thessaloniki, 54124 Thessaloniki, Greece; 4 Department of Animal Science, University of Thessaly, 41110 Larissa, Greece

**Keywords:** bone defect, bone healing, bone fracture, bone substitute, mesenchymal stem cells, nanocrystalline hydroxyapatite, sheep, stromal vascular fraction

## Abstract

**Simple Summary:**

This is the first study in which the efficacy of fresh autologous stromal vascular fraction from adipose tissue was employed in enhancing long bone healing. The objective of the study was to evaluate the benefits of using biomaterial for the treatment of segmental bone defect, employing sheep as the animal model. Bone defects were created and various biomaterials (nHA paste, autogenous bone graft mixed, stromal vascular fraction obtained from adipose tissue of the animals) were used on their own or in combination. Post-operatively, the animals were evaluated clinically and by using imaging techniques. It is concluded that the lumbosacral region was an attractive site for harvesting adipose tissue, the use of stromal vascular fraction contributed to faster rehabilitation post-operatively and stromal vascular fraction significantly enhanced bone formation. In general, the results indicated an osteogenic potential of stromal vascular fraction comparable to the gold standard autologous bone graft.

**Abstract:**

The objectives of the present study were to evaluate (a) the feasibility of using stromal vascular fraction (SVF) and nanocrystalline hydroxyapatite (nHA) paste in combination for the treatment of segmental bone defect, (b) the quality of the callus produced, (c) the potential improvement of the autograft technique, and (d) the direct comparison of the biomaterial to the use of autogenous cancellous bone. Unilateral, segmental mid-diaphyseal bone defect was created on the right metatarsus of skeletally mature sheep animals (*n* = 24) under anesthesia (D0). Residual segments were stabilized by stainless-steel plates and appropriate screws. Defects were managed as follows: group A: use of nHA paste to filling, group B: use of autogenous bone graft mixed with nHA bone paste, placed in defect, group C: use of SVF mixed with nHA bone paste injected into defect, group D: use of bone graft and SVF with nHA paste before apposition in bone defect. SVF had been previously isolated from adipose tissue of the animals intra-operatively after digestion with collagenase solution and neutralization. Animals were evaluated clinically and by X-raying and ultrasonographic examination of the defect, at regular intervals, until D90. Ultrasonographic assessment performed along the length of the defect included calculation of the length of the bone defect and assessment of vascularization. SVF was successfully isolated from group C and D animals, with the average yield being 1.77 × 10^6^ cells. The comparison of clinical scores (based on the ‘Kaler scale’) on each post-operative day indicated significant differences between the four groups on D1 to D30 (*p* < 0.01); the median clinical score within group A was 2.5 for D1-D30 and 1 for the entire period; respective scores for other groups were 1.5 (*p* = 0.07) and 0 (*p* = 0.033). Differences in radiographic assessment scores were significant for scores obtained on D60 (*p* = 0.049) and D90 (*p* = 0.006). There was a significant difference between the four groups in the length of the bone defect, as assessed ultrasonographically, for the entire length of the study; median values were 8, 8.5, 6, and 8 mm for groups A, B, C, and D, respectively (*p* = 0.008). There was a significance in the differences between median scores obtained during the histopathological examination: 2, 11, 13.5, and 12 for group A, B, C, and D (*p* = 0.022). There was an inverse correlation between the overall scores of histopathological evaluations and the length of the bone defect (observed on D90) (*p* < 0.0001) and a correlation between the overall scores and the radiographic assessment scores (obtained on D90) (*p* < 0.0001). This is the first study in which the efficacy of fresh autologous Stromal Vascular Fraction (SVF) from adipose tissue in enhancing bone healing in a long, weight-bearing, diaphyseal bone was evaluated. It is concluded that the lumbosacral region was an attractive site for harvesting adipose tissue, the use of SVF contributed to faster rehabilitation post-operatively, and SVF significantly enhanced bone formation; in general, the results indicated an osteogenic potential of SVF comparable to the gold standard autologous bone graft.

## 1. Introduction

Bone structure and function restoration subsequent to traumatic or pathological conditions usually occur uneventfully by secondary bone healing. Necessary elements for this include osteogenic cells, osteoconductive matrix, osteoinductive stimulus, mechanical stability, and adequate vascular supply [1,2,3]. However, in cases of delayed union or nonunion, multi-fragmentary fractures, bone defects, or necrosis due to osteomyelitis and arthrodesis, bone grafting would be recommended to enhance bone healing. An ideal graft would deliver osteogenic cells directly, would stimulate differentiation of mesenchymal stem cells, and would provide a matrix for new bone ingrowth and augment neo-angiogenesis [4,5,6,7].

Currently, autogenous cancellous bone grafting is considered to be the gold standard owning osteogenic, osteoinductive, and osteoconductive properties and rapid incorporation [3,8,9,10,11]. However, autograft use complications are reported in up to 40% of cases, including limited availability and donor site morbidities, e.g., pain, seroma formation, iatrogenic fracture, post-operative bleeding, and increased time under general anesthesia [8,12,13].

The constantly increasing need for bone grafts coupled with the various limitations regarding the use of autologous bone graft have urged a need to develop alternative bone substitutes. Such materials are osteoconductive and promote bone apposition on their surface functioning as a three-dimensional scaffold supporting vascular and cellular ingrowth, thus leading to new bone formation [5,14,15,16,17]. Relevant advantages include unlimited supply, easy sterilization, and storage [18]. Among these, nanocrystalline hydroxyapatite (nHA) paste can be used as a scaffold due to its biocompatibility, rapid biodegradation, prompt osteogenesis and neoangiogenesis, and larger bone coverage in bone defects than ceramic hydroxyapatite [19,20,21]. nHA paste is known as an osteoconductive bone substitute, also with osteoinductive properties in vivo, as it has been found to stimulate the host mesenchymal stem cells to differentiate into osteoblasts [22,23,24].

Recent research has focused on bone-regenerative therapies, including combinations of multipotent mesenchymal stem cells (MSCs), due to their osteogenic and osteoinductive properties with bone substitutes. After the initial recovery from bone marrow (bone marrow stromal cells), MSCs have been isolated from many other sources, with the adipose tissue serving as the best alternative (adipose-derived stem cells, ASCs) due to its abundance in the body, easier and safer collection, and low morbidity of patients [25,26]. First, Zuk et al. [27] showed that adipose tissue contains ASCs in stromal vascular fraction and reported their osteogenic capacity [27,28]. Adipose tissue can yield up to a 300- to 500-fold greater number of MSCs than bone marrow [25,29,30,31,32]. ASCs were reported to have higher osteogenic potential than bone marrow stromal cells under osteogenic differentiation conditions [33]. They may be cultured in vitro to the desirable differentiation or, instead, a heterogeneous cell fraction can be concentrated from adipose tissue harvest, known as Stromal Vascular Fraction (SVF).

SVF is a heterogeneous combination of ASCs, endothelial cells, endothelial precursor cells, pericytes, T regulatory cells, macrophages, mast cells, smooth muscle cells, and pre-adipocytes, thus possessing not only osteogenic and osteoinductive ability, but also immunomodulatory, anti-inflammatory, and angiogenic capacity [34,35]. Indeed, 2% to 10% of the mononuclear cells of SVF were reported to be ASCs [36]. The numerous cell types in SVF have distinct contributions to its regenerative capabilities through immunomodulatory effects, cell survival properties, controlled inflammation, differentiation capacity, and extracellular matrix and expression of genes associated with healing and angiogenesis, including several growth factors [37,38]. The multipotent cells within the SVF attach very fast to scaffold material, multiply and differentiate towards bone-forming cells rapidly, or increase the paracrine effect to the local cells toward proliferation and differentiation [39]. Another advantage of SVF is that it is more easily acquired, without a need for cell separation or culture and, therefore, and can be applied intra-operatively, thus minimizing the risks induced by culturing cells [34,39,40,41].

There are various comparative studies showing improved outcomes with SVF in terms of promoting cartilage and subchondral bone regeneration and formation of new cartilage matrix [42,43]. These properties are attributed to the varying cell populations in SVF, as highlighted above, which cooperate and stimulate mesenchymal cell activity through paracrine signaling better than ASCs alone, confirming interactions between the various cell populations in SVF and the host microenvironment [37]. Moreover, growth factors and endothelial precursor cells have been shown to improve healing in bone defects [44,45]; further, a synergistic effect on angiogenesis and osteogenesis has been reported when a combination with MSCs was used [46].

The first report of the clinical use of SVF for bone healing referred to a child with widespread calvarial defects after multifragment calvarial fractures and infection where SVF with bone graft achieved skull reconstruction after a single surgical procedure [47]. Since then, research has focused on the use of autologous SVF for bone defects healing in animal models, reporting encouraging results in flat bones [48,49,50,51] and defects created on femoral condyles [41,52]. Feasibility, safety, and potential efficacy for bone healing can be attributed to the differentiation of the ASCs within SVF towards bone-forming cells, the increased paracrine effect of the SVF, and the high angiogenic potential [39,53]. Recent research comparing the bone-regenerative potential of SVF and ASCs has indicated improved osteoinductive ability and larger callus formation of SVF than in vitro cultured ASCs due to cell heterogenicity [41,54].

To the best of our knowledge, there is a paucity of information regarding the potential enhancement of healing of diaphyseal long bone defects through the combined use of SVF and nHA paste.

The objectives of the present study were to evaluate (a) the feasibility of using stromal vascular fraction and nanocrystalline hydroxyapatite paste in combination for the treatment of segmental bone defect, (b) the quality of the callus produced, (c) the potential improvement of the autograft technique, and (d) the direct comparison of the biomaterial to the use of autogenous cancellous bone.

## 2. Materials and Methods

### 2.1. Experimental Overview, Animals, Examinations, and Samplings

A total of 24 female multiparous sheep, 4 to 5 years old (i.e., skeletally mature), were enrolled in this study and randomly allocated into four equal groups (by using an electronic random number generator (www.randomresult.com, accessed on 25 November 2020)). The median (interquartile range) bodyweight of the animals was 70 (22.5) kg (*p* = 0.99 between groups). Animals were housed at the experimental facilities of the faculty, allowing approximately an area of 6 to 7 m^2^ per animal, and standard health management procedures (vaccinations, anthelmintic treatments) were followed.

In all the animals, under general and epidural anesthesia, unilateral, segmental mid-diaphyseal bone defect was created in the right metatarsal bone. The defect created was stabilized by using orthopedic bone plates and screws and filled. Details of the experimental groups are in Table 1.

Clinical examination and imaging assessments of animals were performed three days before surgery (D–3), as well as on D0 before the actual surgical procedure. Blood samples were also collected. Post-operatively, similar examinations were also carried out at regular intervals for up to D90 (Section 2.5). Finally, animals were euthanized 13 weeks post-surgery for a detailed pathological examination.

### 2.2. Operation Procedures: Anaesthetic and Analgesic Protocols, Surgical Methodologies

Premedication included medetomidine (dose rate: 10 μg kg^−1^ bodyweight; Sedator; Eurovet Animal Health, Bladel, The Netherlands), midazolam (dose rate: 0.2 mg kg^−1^ bodyweight; Dormicum; Roche Pharma, Basel, Switzerland), and morphine (dose rate: 0.3 mg kg^−1^ bodyweight; Morfina cloridrato; Molteni Pharmaceutici, Scandicci, Italy).

Epidural anesthesia was then induced by lidocaine hydrochloride (dose rate: 2.0 mg kg^−1^ bodyweight; Xylozan 2% *w*/*v*; Demo, Athens, Greece) and morphine (dose rate: 0.1 mg kg^−1^ bodyweight), which were administered via the lumbosacral space.

At that stage, in animals in groups C and D, adipose tissue was collected. Hair at the lumbosacral region was clipped and the area at the base of the tail was aseptically prepared. Skin and subcutaneous tissue were excised, and fat tissue was cut out en-block for harvesting Stromal Vascular Fraction (SVF) (Section 2.3.1). Subcutaneous tissue and skin were closed in a routine suturing pattern.

Subsequently, general anesthesia was induced and established with intravenous administration of propofol (dose rate: 1.0–2.0 mg kg^−1^ bodyweight; Propofol/Lipuro; Braun Melsungen, Melsungen, Germany) and ketamine (dose rate: 1.5 mg kg^−1^ bodyweight; Ketaset; Zoetis, Parsippany-Troy Hills, NJ, USA). Anesthesia was maintained with isoflurane (1.5–2.0% in oxygen through a semi-closed circuit; Vetflurane; Virbac, Carros, France).

At that stage, in animals in groups B and D, bone graft was harvested. The iliac crest was appropriately prepared; a curved skin and subcutaneous tissues incision was made along the iliac crest. Bone graft was harvested with a bone spoon after drilling holes to the cortice. The graft was maintained in a sterile syringe filled with blood until it was used appropriately. Subcutaneous tissue and skin were closed in a routine suturing pattern.

Next, in all animals, a bone defect was created. The animal was positioned on the operating table in right lateral recumbency with the left hind leg abducted and retracted caudally; the right hind leg was aseptically prepared. A skin and fascia incision was made on the medial surface of the metatarsus and the periosteum was carefully retracted at the middle third of the diaphysis of the metatarsus. The bone plate (DCP 3.5 mm 10-hole; Veterinary Instrumentation, Sheffield, United Kingdom) was initially positioned and bent appropriately. The central holes were left empty, demarcating the position of the defect, whereas the remaining adjacent screw holes (Veterinary Instrumentation) were prepared. The plate was then removed and, under continuous saline irrigation, a 15 mm-long segmental bone defect (ratio segmental length: bone diaphysis diameter = 1:1) was created at the middle third of the diaphysis of the bone by using an oscillating bone saw (Veterinary Instrumentation). Next, the bone plate was secured on the bone with four screws on each side of the defect. Finally, the bone defect was filled with the respective biomaterial (Table 1, Section 2.3.2.). The subcutaneous and skin layers were closed in a routine suturing pattern.

Post-operative analgesia was provided by intravenous injection of meloxicam (dose rate: 0.3 mg kg^−1^ bodyweight; Metacam; Boehringer Ingelheim Animal Health, Ingelheim am Rhein, Germany) immediately after the end of the surgical procedure and on D3.

### 2.3. Intra-Operative Procedures

#### 2.3.1. Harvesting of Adipose Tissue and Isolation of Stromal Vascular Fraction

Adipose tissue was collected from each animal in order to harvest autologous SVF, which was performed as previously described [27,55]. In brief, 15 mL from the adipose tissue collected were washed repeatedly in phosphate-buffered saline (PBS) (GibcoTM, Thermo Fisher, Carlsbad, CA, USA) to remove erythrocytes and tissue debris and then centrifuged at 300× *g* at 20 °C for 5 min in order to separate the wash from the fat layer. The PBS wash was aspirated and discarded and the remaining material (fat layer and cell pellet) was incubated with 0.1% solution of collagenase I (GibcoTM, Thermo Fisher) for 60 min at 37 °C, at which time-point Dulbecco’s modified Eagle medium (Nutrient Mixture F-12, DMEM/F-12; Sigma-Aldrich, St. Louis, MO, USA) was added to neutralize the collagenase. Subsequently, SVF pellets were obtained by centrifugation at 300× *g* at 20 °C for 5 min, followed by discarding the supernatant and resuspension in PBS; the procedure was repeated twice. The solution thus obtained was used in the surgery in groups C and D (Section 2.3.2). In order to evaluate the concentration of SVF in the final solution, a quantity of 10 μL of the cell pellet was mixed with 10 μL of trypan blue (GibcoTM, Thermo Fisher) and the combination was placed onto the Neubauer plate, where SVF cells were observed and counted in all four outer quadrants by the following formula: SVF cells per mL of adipose tissue = $\frac{\begin{array}{c} \mathrm{number}\;\mathrm{of}\;\mathrm{cells}\;\mathrm{counted}\;\mathrm{on}\;\mathrm{the}\;\mathrm{plate} \end{array}}{4}\times$ 2 × 10,000.

#### 2.3.2. Filling of the Bone Defect

The material used for filling the bone defect was prepared before use, whilst the respective animal was in the surgical procedure. For animals in group A, sterile bone nanocrystalline hydroxyapatite (nHA) paste (38% nanocrystalline hydroxyapatite in water) (Nano HA Bone Paste; Veterinary Instrumentation, Sheffield, UK) was added. For animals in group B, a mixture (1:1 in weight) of nHA paste and bone graft [56] was added. For animals in group C, a mixture (1:1 in volume) of nHA paste and the SVF solution was added. Finally, for animals in group D, a mixture (1:1:1 in volume) of nHA paste, bone graft, and the SVF solution was added in the defect. In all cases, the mixtures were prepared and left for 30 min before implantation, as described previously [53,56,57]. In all animals, the total volume of the material used for filling the defect varied from 2.2 to 2.4 mL.

### 2.4. Post-Operative Management

An external coaptation (Orthopaedic Casting Tape, Henry Schein, Gillingham, UK), extending downwards from the distal tibia, was applied on the operated part of the limb post-surgically. The cast was open on the caudal aspect to facilitate regular bandaging changes, clinical assessment, and imaging of the limb.

Additional pharmaceutical treatment included administration of long-acting oxytetracycline (dose rate: 20 mg kg^−1^ bodyweight; Tenalin, Ceva Sante Animale, Libourne, France) at surgery and on D3 and a combination of lincomycin and spectinomycin (dose rate: 5 and 10 mg kg^−1^ bodyweight; Alfasan, Woerden, The Netherlands) daily until D5.

Post-operatively, the animals were allowed to move freely as tolerated.

### 2.5. Clinical and Paraclinical Examinations

#### 2.5.1. Clinical Examination

Post-operatively, the animals were examined on D1, D2, D3, D10, and every 10 days thereafter until D90.

The animals were examined clinically by following established methodologies [58] and for abnormal behavior. A detailed orthopedic examination was performed, with special attention paid to their legs and feet [59]. Limb alignment and stability of the fractured site were assessed in detail. The surgical trauma was examined for evidence of abnormalities.

#### 2.5.2. Radiographic Examination

Post-operatively, standard digital craniocaudal and mediolateral X-ray images were obtained on D1 and then on D30 and every 30 days thereafter until D90.

#### 2.5.3. Ultrasonographic Examination

Post-operatively, ultrasonographic examinations were carried out on D1, on D10, and every 10 days thereafter up to D60 and then every 15 days until D90. The examination involved longitudinal scans using ultrasonographic equipment (MyLab^®^ 30; ESAOTE SpA, Genova, Italy) with a linear transducer (frequencies available 7.5–12.0 MHz). The scans were performed along the length of the lateral surface of the metatarsus and imaged the bone from the proximal to the distal segment, including the defect.

B-mode imaging was performed initially in order to assess the bone defect, followed by color Doppler imaging to evaluate callus vascularity. The work was performed with the animal in the standing position. The following settings were used for the assessment: frequency 10 MHz, imaging depth of 4 to 5 cm, and pulse repetition frequency 14 KHz.

The length of the bone defect was calculated by the equipment’s software; after pointing out the boundaries of the defect, the length was calculated. Vascularization in the defect and the surrounding soft tissues, as well as vascular signal intensity, was evaluated.

#### 2.5.4. Histological Examination

After euthanasia, a 3 cm-long segment of the metatarsal bone, immediately beneath the bone plate, was excised and placed into 10% neutral buffered formalin. The tissue samples were processed for histological examination by applying standard histopathological procedures. In brief, formalin fixation, decalcification, excision of longitudinal section, dehydration, and embedding in paraffin were performed sequentially. Finally, 4 to 6 μm sections were stained with hematoxylin and eosin.

### 2.6. Data Management and Analysis

#### 2.6.1. Data Management

During clinical examination, for the assessment of motility of the animal and potential presence of lameness, a standardized system of examination was used and a score from 0 (best) to 6 (worst) was assigned [60] (Table S1).

During radiographic examination, findings referring to bone formation, union, and remodeling were scored according to the modified Lane and Sandhu scoring system from 0 (worst) to 10 (best) [61] (Table S2).

During ultrasonographic examination, vascularization observed at the area of the defect and around it was scored at a scale of 0 (no vascularization) to 3 (maximum vascularization) [62,63] (Table S3). Three images were taken on each examination occasion and each of them was scored separately, and then the median value of the three scores was considered.

During histopathological examination, the sections were evaluated and scored on a 0 (worst) to 15 (best) overall score by using a standardized assessment system (graft incorporation, bone cortex activity, callus and new tissue formed, bridging of the edges of the defect, periosteum vascularity) [64] (Table S4).

#### 2.6.2. Statistical Analysis

Data were entered into Microsoft Excel and analyzed using SPSS v. 21 (IBM Analytics, Armonk, NY, USA). Initially, a basic descriptive analysis was performed.

Comparisons between groups were made by using the Kruskal–Wallis test or Pearson’s chi-square test. The potential associations between results obtained by the various techniques employed for assessments were evaluated by using Spearman’s analysis of correlation. Repeated measures mixed-effect linear regression was used to evaluate the significance of changes throughout the study.

In all analyses, statistical significance was defined at *p* < 0.05.

## 3. Results

### 3.1. Outcome of Surgeries

The isolation of Stromal Vascular Fraction (SVF) (performed always within 90 min, with a mean yield of 1.77 × 10^6^ ± 0.09 × 10^6^ mononuclear cells mL^−1^), the preparation of appropriate biomaterial employed on each animal into the study, and the surgical procedures were completed uneventfully on all occasions.

### 3.2. Clinical Examination

Clinical examinations and the orthopedic assessments performed before surgery did not reveal abnormalities in the animals. All animals were assigned scores of 0 in the relevant scale.

The comparison of clinical scores on each post-operative day indicated significant differences between the four groups on D1 to D30 (*p* < 0.01). Thereafter, no significant differences were seen between groups (*p* > 0.94). No abnormalities were found in the surgical trauma of any animal in any group in the study.

The median (interquartile range) clinical score within group A was 2.5 (2) for the period D1 to D30 and 1 (2.3) for the entire post-operative period; respective scores for the other three groups were 1.5 (3) (*p* = 0.07) and 0 (1.3) (*p* = 0.033). There was no difference in the slope of the progressive decrease of clinical scores between the four groups: −0.026 ± 0.010 for A and –0.021 ± 0.010 for B, C, and D (*p* = 0.74). The results of the post-operative assessment of lameness are summarized in Table 2.

### 3.3. Imaging Examination

#### 3.3.1. Radiographic Examination

**Figure 1:**
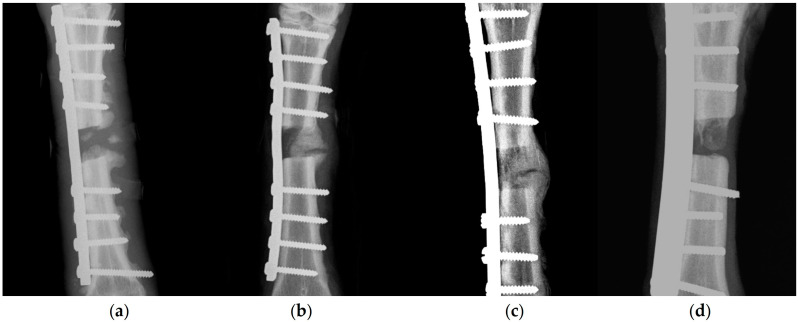
X-rays of metatarsal bone of sheep, taken on 90 days after creation of bone defect and implantation of various biomaterials in there for (**a**) group A (score 3/10), (**b**) group B (score 6/10), (**c**) group C (score 7/10), and (**d**) group D (score 6/10) (scores given according to Lane and Sandhu [61] (Table S2)).

**Figure 2:**
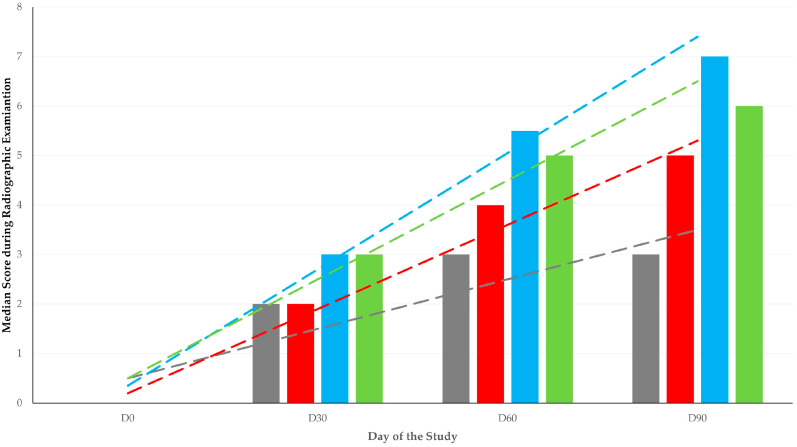
Scores during radiographic examination in four groups (A: grey, B: red, C: blue, D: green) of sheep for 90 days after creation of bone defect and implantation of various biomaterials in there (dashed lines show respective trendlines; slopes A: 0.033 ± 0.011, B: 0.057 ± 0.006, C: 0.078 ± 0.008, D: 0.067 ± 0.011; *p* = 0.030 between A and C, *p* > 0.10 for all other comparisons).

Overall, there were no significant differences between the four groups in the radiographic assessment scores; median (interquartile range) scores were 2 (2.3), 3 (4.5), 4 (5.5), and 4 (3.8) for groups A, B, C, and D, respectively (*p* = 0.09) (Figure 1). When scores were considered on a daily basis, differences were seen for scores obtained on D60 (*p* = 0.049) and D90 (*p* = 0.006) in two orthogonal views (Table 3, Figure 2).

Overall, there was also a clear inverse correlation between the clinical scores and the radiographic assessment scores (*r_s_* = −0.477, *p* < 0.0001).

#### 3.3.2. Ultrasonographic Examination

**Figure 3:**
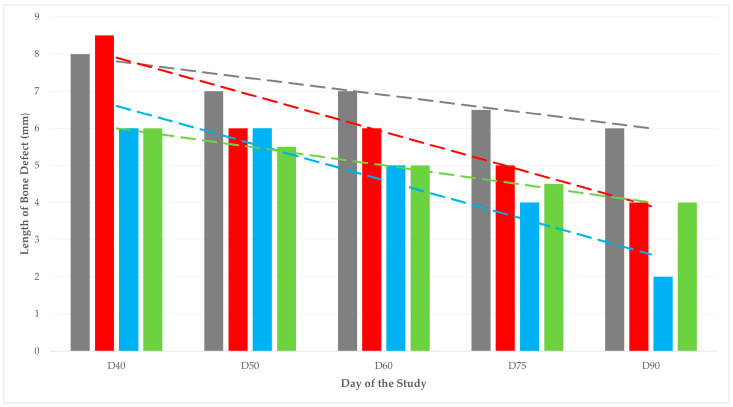
Length of bone defect in four groups (A: grey, B: red, C: blue, D: green) of sheep during 40 to 90 days after the creation of bone defect and the implantation of various biomaterials in there (dashed lines show respective trendlines; slopes A: −0.035 ± 0.007, B: −0.078 ± 0.018, C: −0.082 ± 0.012, D: −0.040 ± 0.002; *p* = 0.011 between A and C, *p* > 0.06 for all other comparisons).

**Figure 4:**
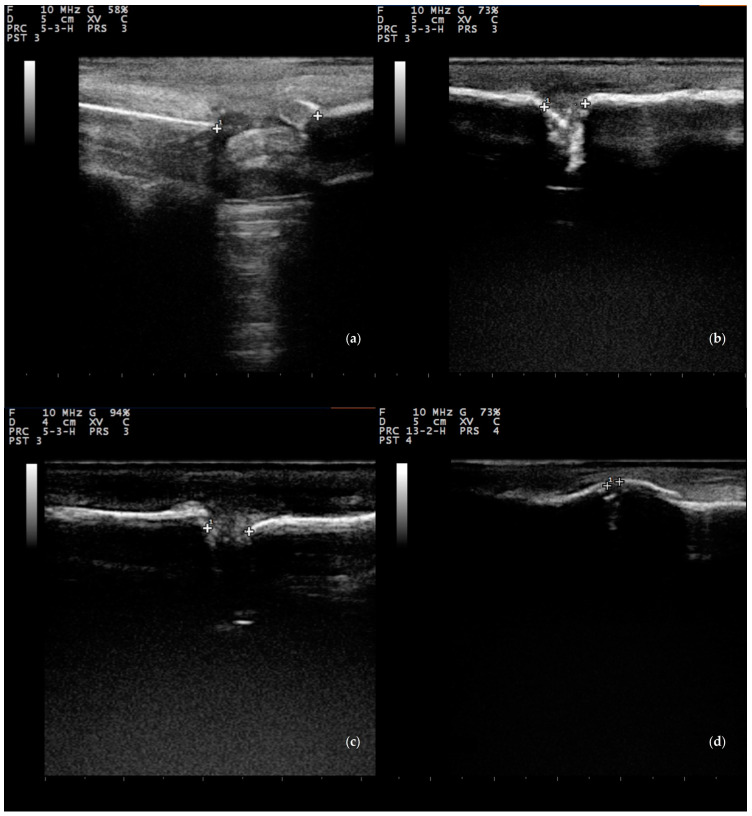
Length of the bone defect, measured ultrasonographically, in group C sheep (**a**) 10 (16 mm), (**b**) 30 (6.5 mm), (**c**) 60 (5 mm), and (**d**) 90 (2 mm) days after the creation of bone defect and the implantation of biomaterials (fresh stromal vascular fraction with nanocrystalline hydroxyapatite paste).

**Figure 5:**
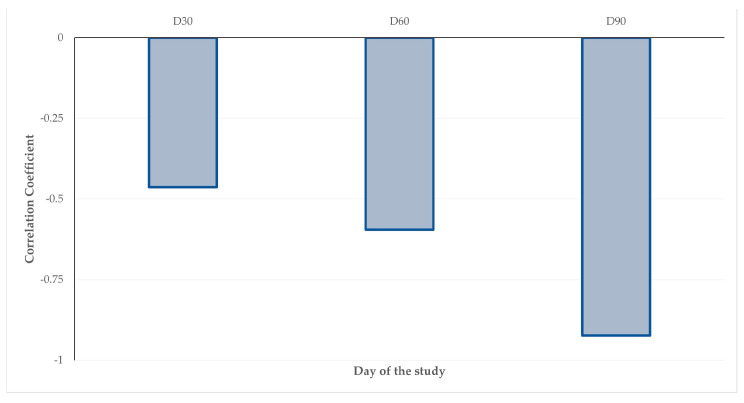
Progressive decrease (D30, D60, D90) of the correlation coefficient between the length of the bone defect and the radiographic assessment scores.

Overall, there was a significant difference between the four groups in the length of the bone defect for the entire length of the study; median (interquartile range) values were 8 (4), 8.5 (8), 6 (5.5), and 8 (7.8) mm for groups A, B, C, and D, respectively (*p* = 0.008). When scores were considered for the period D1 to D30, no significant differences were seen (*p* = 0.42), whilst on a daily basis, significant differences were seen for scores obtained on D75 (*p* = 0.006) and D90 (*p* = 0.002) (Table 4, Figure 3, Figure 4 and Figure 5).

There was no difference in the vascularization scale between the four groups, neither overall (*p* = 0.44) nor at any day of the study (*p* > 0.44) (Table 5). However, a significantly higher proportion of animals in groups B, C, and D achieved a score of ‘0′ on D75 and D90 (in >83% of cases) than in group A (in 25% of cases) (*p* = 0.0003). Overall, there was a clear positive correlation between the length of the bone defect and the vascularization scale (*r_s_* = 0.675, *p* < 0.0001).

### 3.4. Histological Examination

There was a significance in the differences between median (interquartile range) scores obtained during the histopathological examination: 2 (1), 11 (4.5), 13.5 (4.8), and 12 (4.5) for group A, B, C, and D, respectively (Figure S1) (*p* = 0.022).

**Figure 6:**
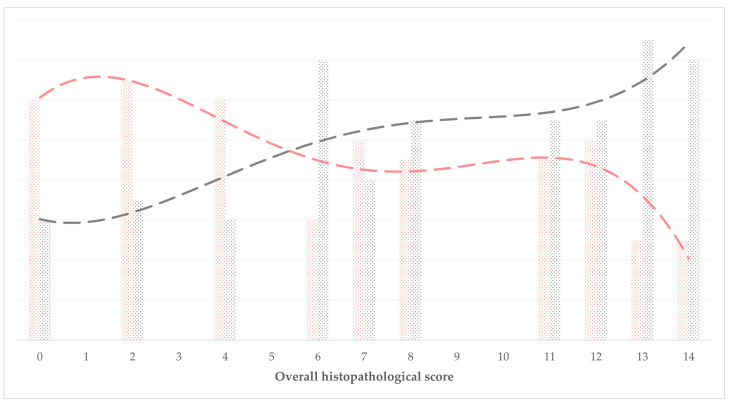
Association of the overall histopathological scale (*x* axis) with corresponding median D90-values of bone defect length (pink colored) or radiographic assessment score (grey colored) (dashed lines show respective trendlines).

When the overall scores of histopathological evaluation were taken into account independently of groups, there was an inverse correlation between these and the length of the bone defect (observed on D90) (*r_s_* = −0.784, *p* < 0.0001), a correlation between the overall scores and the radiographic assessment scores (obtained on D90) (*r_s_* = 0.783, *p* < 0.0001), but not with the vascularization scale (obtained on D90) (*r_s_* = −0.226, *p* = 0.31). Indeed, with increasing overall scale of the histopathological evaluation, there was a clear decrease of the length of the bone defect and increase of the radiographic assessment scores (Figure 6).

## 4. Discussion

### 4.1. Animal Model Employed

In order to evaluate successful application and possible advantages of Stromal Vascular Fraction (SVF) in long bone defects, sheep were used as the animal model. Adult sheep present similarities with humans and other veterinary patients in bodyweight, complexity of bone architecture, and bone remodeling [65,66]. Moreover, sheep are docile and easy to handle animals, thus offering advantages during their handling. A critical-sized defect in the metatarsus was created for the study because it is a straight, weight-bearing bone [66]; moreover, the lack of musculature facilitates a surgical approach, whilst the reduced blood supply can contribute to delayed fracture healing (thus introducing a difficult-case scenario). Indeed, the distal selection of the ostectomy site can contribute to a risk of non-union [67].

Stem cells can be a useful tool in tissue regeneration. In order to be applied in practice, mesenchymal stem cells should be easily obtained, with little cost and minimally handled to avoid potential contamination, risk of carcinogenesis or any other adverse effect on their functional characteristics [68]. Thus, the intra-operative isolation of SVF offers a critical advantage and excludes the need for a second operation, thus, minimizing the cost and morbidities associated with repetitive surgical procedures. Further, its use may offer an advantage in emergency cases when there is no time for a cell culturing procedure. Moreover, Bonab et al. [69] have demonstrated that in series of subcultures, mesenchymal stem cells start to show a decreased potential due to a decrease on telomerase activity, resulting in cellular senescence [69].

During the surgical phase of the study, the procedure was feasible when performed by veterinarians with surgical experience. This was confirmed by the successful isolation of SVF cells and the return to the operation theatre for use within 90 min of collection, as described in the literature [70]. Given that this is an average time-period necessary for fracture reduction and fixation in animals, it is suggested that a surgeon might possibly harvest the fat before the approach to the fracture site; this can then be processed, concurrently with the surgical orthopedic work so that it is finally implanted into the fracture site before soft tissue closure. Benefits of SVF compared to autogenous bone graft/bone marrow harvesting include painless collection and availability in large quantities due to adipose tissue abundance [71,72], good quality even in patients with osteoporosis [52], and larger numbers of adipose-derived stem cells (ASCs) [25,29,30,31,32]. Moreover, all the surgical and clinical procedures were completed uneventfully. The findings are in accordance with previous reports regarding safety in using SVF cells in clinical situations [52,73,74,75].

A previous study carried out in sheep found a mean number of 1.88 × 10^6^ cells g^−1^ SVF in the adipose tissue [40]. That one is the only study in an ovine model and involves adipose tissue collected from the cervicothoracic region for osteoarthritis treatment. In the present study, under the same conditions and by using a similar protocol, the isolation of SVF was comparable (on average, 1.77 × 10^6^ cells mL^−1^) derived from the lumbosacral region. Additionally, this donor site offers the advantage of effective and painless adipose tissue collection under epidural anesthesia with no need for administration of general or local anesthesia, which has been reported to negatively affect cell viability and cell growth rate of ASCs in vitro [76]. A comparative study in dogs revealed better results in terms of viable cells of adipose tissue collected at the thoracic wall caudally to the scapula and inguinal sites than the falciform ligament [77] but had not considered the lumbosacral region as an option. The cell yield in the vascular stromal fractions collected from the inguinal region of dogs was found to be on average 4.2 × 10^5^ viable cells g^−1^ of adipose tissue [78]. Finally, and according to the literature, in human SVF, nucleated cells range from 0.5 × 10^6^ to 2 × 10^6^ cells g^−1^; 1% to 10% of these nucleated cells were reported to be ASCs [35,36]. Overall, and based on the outcomes of the present study, in sheep the lumbosacral region appears to serve as an attractive alternative site for adipose tissue harvesting.

### 4.2. Outcomes of the Procedure

The comparison of locomotion scores indicated significant differences between groups starting on D3 and up to D30. During that period, animals in groups B, C, and D showed significantly better locomotion ability and, therefore, a faster rehabilitation than group A animals. The contribution of SVF on pain reduction and motility improvement has been widely investigated for the treatment of osteoarthritis both in human and animal species. For example, the injection of autologous SVF in osteoarthritic joints resulted in clinical improvement in pain and range of motion [73,79,80,81,82,83,84,85]. With regard to animal studies, dogs with hip joint osteoarthritis showed improvement in pain and gait scoring scale compared after treatment with SVF and PRP [86]. The reduction in pain can be attributed to the anti-inflammatory properties of SVF cells, possible modulation on T cells and B cells, expression of anti-inflammatory mediators, and secretion of various other soluble factors [26,38,87]. Similarly, autogenous bone graft, as used in animals of Group B, has been found to provide cytokines and growth factors, enabling them to induce early clinical restoration as well [88]. Results of the present study have indicated that the effect of SVF on pain reduction and faster mobilization of the limb could also be beneficial for the fracture management of long bones.

Among imaging assays, radiography remains the most common method for the assessment of fracture union. In a systematic review of 122 fracture studies using imaging criteria, X-rays were used in 98% of these, although often with no use of valid and reliable radiographic measures of union [89]. In the present study, the Modified Lane and Sandhu radiological scoring system was used to objectively assess the radiological findings, as this takes into account bone-related variables (bone formation, union proximally and distally and remodeling) [61]. Differences between groups were seen on D60 and on D90. SVF’s osteogenic and osteoinductive ability, as well as its successful incorporation with acellular nHA paste, reflect on the significantly higher proportion of bone formation at the defect and union, both proximal and distal, in animals treated with SVF and nHA paste. The relatively low radiographic assessment scores and the absence of differences on antecedent measurement of the study period was an expected result since radiography is a less sensitive assay in evaluation of soft callus in the early stages of bone formation. Nevertheless, ultrasonography can be a more sensitive and accurate means for detection of early phases of the callus and its progression to bridging new bone formation [90,91,92,93]. Indeed, the use of B-mode ultrasonography has enabled us to estimate the length and the bridging potential of the bone defect during healing as osteoid was deposited. Most animals showed a negligible increase in the length of the bone defect on D10, which was subsequently decreased. This was an expected finding due to the temporarily impaired blood perfusion and bone resorption at the borders of the ostectomy site. However, significant differences at the length of the bone defect between the four groups were obtained on D75 and D90. Animals in group C showed a significant reduction in the length of the bone defect due to the formation of callus bridging the defect, whereas animals in groups B and D also had reduced length compared to group A. The present results are in accord with similar studies in rats, in which the use of allogenic SVF on critical-sized femoral bone defects of rats resulted in bridging and partial mineralization [52]; it is noted that, at that study, unstable pseudarthrosis formation was also recorded in the acellular hydroxyapatite microgranules filled defects [52].

In the early stages of bone healing, blood perfusion of the fracture site and the surrounding soft tissues is critical, as it delivers oxygen and nutrients to the site of injury. Ultrasonographic studies in both animals and people have suggested the use of the Color or Power Doppler for the time-dependent evaluation of development and regression of vascularization during fracture healing in long bones [62,94,95,96,97]. In the present study, vessel development and density inside the callus ranged within normal values in all groups throughout the whole study period, suggesting that the materials used to fill the defect created a friendly environment for the vascularization and blood flow at the fracture site. However, the significantly lower detection of blood flow in animals which had received SVF and/or bone graft, favored a more uneventful healing process in those groups and the potential development of fibrous callus formation and the delayed union, or even a non-union, in animals that received solely nHA paste.

The findings of the histopathological examination have confirmed the results of the clinical and imaging examinations. The full incorporation of the SVF and bone graft into new bone, osseous callus formation and advanced bridging of the bone defect was recorded in these groups. The combination of SVF with acellular nHA paste was at least as effective as that of the bone graft combination and significantly more effective than the use of nHA paste alone.

Finally, the progressive reduction in correlation coefficient between the length of the bone defect and the radiographic assessment scores indicate an enhanced reliability of ultrasonography as a sensitive and accurate means for the detection of early phases of bone formation, while radiographic imaging appears to become a more accurate technique with the advancement of time.

## 5. Conclusions

This is the first study in which the efficacy of fresh autologous Stromal Vascular Fraction (SVF) from adipose tissue in enhancing bone healing in a long, weight-bearing, diaphyseal bone was evaluated. All essential procedures (from fat graft and/or bone graft harvesting to the implantation of the material at the site of the bone defect) were successfully performed in a one-step surgical procedure. Autologous SVF was compared with autologous cancellous bone graft for the first time, resulting in encouraging outcomes and thus offering an attractive alternative in bone bioengineering.

The results of the present study indicated the feasible use of fresh, autologous SVF to enhance bone healing in a critical diaphyseal bone defect, in a load-bearing bone in an ovine model. The procedure was completed safely, without any local or systemic adverse effects for a period of 3 months post-operatively. SVF was successfully isolated in an adequate numbers of cells within a short period of time, in a one-step surgical procedure. The lumbosacral region was an attractive site for harvesting adipose tissue. The use of SVF contributed to faster rehabilitation post-operatively and indeed SVF significantly enhanced bone formation. In general, the results indicated an osteogenic potential of SVF comparable to the gold standard autologous bone graft.

## Figures and Tables

**Table 1:** Details of the experimental design.

Group	Collection of Adipose Tissue Intra-Operatively	Collection of Bone Graft Intra-Operatively	Filling of the Defect ^1^
A (*n* = 6)	No	No	nHA paste
B (*n* = 6)	No	Yes	Autologous BG with nHA paste
C (*n* = 6)	Yes	No	Fresh SVF with nHA paste
D (*n* = 6)	Yes	Yes	Fresh SVF, autologous BG with nHA paste

^1^ nHA: nanocrystalline hydroxyapatite, BG: bone graft, SVF: stromal vascular fraction.

**Table 2:** Median (interquartile range) clinical scores ^1^ in four groups of sheep before and for 90 days after the creation of bone defect and the implantation of various biomaterials in there.

Group	Day of the Study													
	D–3	D–1	D1	D2	D3 ^2^	D10 ^2^	D20 ^2^	D30 ^2^	D40	D50	D60	D70	D80	D90
A	0 (0)	0 (0)	4(0)	3 (0)	3 (0)	2 (0)	1 (0)	1 (0)	0 (0)	0 (0)	0 (0)	0 (0)	0 (0)	0 (0)
B	0 (0)	0 (0)	4 (0)	3 (0)	2 (0.8)	1 (0)	0 (0)	0 (0)	0 (0)	0 (0)	0 (0)	0 (0)	0 (0)	0 (0)
C	0 (0)	0 (0)	4 (0)	3 (0)	2 (0)	1 (0)	0 (0)	0 (0)	0 (0)	0 (0)	0 (0)	0 (0)	0 (0)	0 (0)
D	0 (0)	0 (0)	4 (0)	3 (0)	2 (0)	1 (0)	0 (0)	0 (0)	0 (0)	0 (0)	0 (0)	0 (0)	0 (0)	0 (0)

^1^ min. 0 (best)–max 6 (worst); ^2^ *p* < 0.01 between groups on those study days.

**Table 3:** Median (interquartile range) scores ^1^ during radiographic examination in four groups of sheep for 90 days after the creation of bone defect and the implantation of various biomaterials in there.

Group	Day of the Study			
	D1	D30	D60 ^2^	D90 ^2^
A	0 (0)	2 (0)	3 (0)	3 (0.8)
B	0 (0)	2 (1.5)	4 (2.8)	5 (2.8)
C	0 (0)	3 (0.8)	5.5 (1.8)	7 (0.8)
D	0 (0)	3 (0.8)	5 (0.8)	6 (1.5)

^1^ min. 0 (worst)–max 10 (best); ^2^ *p* < 0.05 between groups on those study days.

**Table 4:** Median (interquartile range) length (mm) of the bone defect in four groups of sheep for 90 days after the creation of bone defect and the implantation of various biomaterials in there.

Group	Day of the Study								
	D1	D10	D20	D30	D40	D50	D60	D75 ^1^	D90 ^1^
A	15 (0)	17 (0.8)	11 (0)	10 (1.5)	8.5 (1)	7 (0.8)	7 (1.5)	6.5 (1)	6 (0.8)
B	15 (0)	15.5 (2.5)	13 (2.8)	10.5 (2.5)	8.5 (2.5)	6 (1.5)	6 (1.5)	5 (0.8)	4.5 (1)
C	15 (0)	16 (0.8)	8.5 (3.3)	7 (0.8)	6 (0.8)	6 (0)	5 (0.8)	4 (0)	2 (0.8)
D	15 (0)	14.5 (1.8)	12 (1.5)	9 (2)	6 (1.5)	5.5 (3.3)	5 (3.5)	4.5 (1.8)	4 (0.8)

^1^ *p* < 0.01 between groups on those study days.

**Table 5:** Median (interquartile range) vascularization scale ^1^ in four groups of sheep for 90 days after the creation of bone defect and the implantation of various biomaterials in there.

Group	Day of the Study							
	D10	D20	D30	D40	D50	D60	D75	D90
A	1 (0.8)	2 (0.8)	2 (0)	1 (0.8)	1 (0)	1 (0)	1 (0)	0.5 (1)
B	1 (0.8)	2.5 (1)	1.5 (1)	1 (0)	1 (0)	1 (0.8)	0 (0.8)	0 (0)
C	2 (0)	3 (0)	2 (0.8)	1 (0)	1 (0)	1 (0.8)	0 (0)	0 (0)
D	1 (0)	3 (0.8)	2 (0)	1 (0.8)	1 (0)	1 (0.8)	0 (0)	0 (0)

^1^ min. 0 (no vascularization)–max 3 (maximum vascularization).

## Data Availability

Detailed data are available upon request from the corresponding author. The data are not publicly available, as they form part of the PhD thesis of the first author, which has not yet been examined, approved, and uploaded in the official depository of Ph.D. theses from Greek universities.

## Supplementary Materials

The following supporting information can be downloaded at: https://www.mdpi.com/article/10.3390/ani13182871/s1, Table S1: Presentation of the standardized system of examination employed for the assessment of motility of the animal and potential presence of lameness; Table S2: Presentation of the scoring system employed during radiographic examination for findings referring to bone formation, union and remodeling; Table S3: Presentation of the assessment of vascularization observed by means of power Doppler ultrasonographic examination at the area of the defect and around it; Table S4: Presentation of the standardized histopathological assessment system employed for scaling findings in tissue samples; Figure S1: Histological pictures of metatarsal bone in sheep, 90 days after creation of bone defect and implantation of various biomaterials in there.
