# Supplementary material for: The Use of Stromal Vascular Fraction in Long Bone Defect Healing in Sheep
Source: Animals (Basel). 2023 Sep 9;13(18):2871. doi: 10.3390/ani13182871 (PMC10525334; doi:10.3390/ani13182871)

The Use of Stromal Vascular Fraction in Long Bone Defect Healing in Sheep

Elena I. Pappa, Mariana S. Barbagianni, Stefanos G. Georgiou, Labrini V. Athanasiou, Dimitra Psalla, Dionysios Vekios, Eleni I. Katsarou, Natalia G.C. Vasileiou, Pagona G. Gouletsou, Apostolos D. Galatos, Nikitas N. Prassinos, Dimitris A. Gougoulis, Marianna Aggelidou, Vicky Tsioli, George C. Fthenakis and Aikaterini I. Sideri

Table S1. Presentation of the standardized system of examination employed for the assessment of motility of the animal and the potential presence of lameness [Kaler et al. 2009 [1]].

**Posture and Locomotion**	**Score**						
	0	1	2	3	4	5	6
Bears weight evenly on all four feet	√						
Uneven posture, but no clear shortening of stride		√	√	√	√	√	
Short stride on one leg compared with others		√	√	√	√	√	
Visible nodding of head in time with short stride			√				
Excessive flicking of head, more than nodding, in time with short stride				√	√	√	
Not weight bearing on affected limb when standing				√	√	√	
Discomfort when moving				√	√	√	
Not weight bearing on affected limb when moving					√	√	
Extreme difficulty rising						√	
Reluctant to move once standing						√	
More than one limb affected						√	
Will not stand or move							√

[1] Reference

Kaler, J.; Wassink, G.J.; Green, L.E. The inter- and intra-observer reliability of a locomotion scoring scale for sheep. *Vet. J.* **2009**, *180*, 189–194.

Table S2. Presentation of the scoring system employed during radiographic examination for findings referring to bone formation, union, and remodeling [Lane and Sandhu 1987 [1]].

Score	Description of Findings
	Bone Formation
0	No evidence of bone formation
1	Bone formation occupying 25% of the defect
2	Bone formation occupying 50% of the defect
3	Bone formation occupying 75% of the defect
4	Bone formation occupying 100% of the defect
	Union (proximal and distal evaluated separately)
0	No union
1	Possible union
2	Radiographic union
	Remodeling
0	No evidence of remodeling
1	Remodeling of medullary canal
2	Full remodeling of cortex

[1] Reference

Lane, J.M.; Sandhu, H. Current Approaches to experimental bone grafting. *Orthop. Clin. North Am.* **1987**, *18*, 213–225.

Table S3. Presentation of the assessment of vascularization observed by means of power Doppler ultrasonographic examination at the area of the defect and around it [Risselada et al. 2006, Jeon et al. 2020 [1]].

Score	No. of Vascular Signs Observed	Signal Intensity
0	No signal	No signal
1	1 – 5	Red / purple color
2	6 – 10	Orange color
3	> 10	Yellow color

[1] References

Risselada, M.; Kramer, M.; Saunders, J.H.; Verleyen, P.; Van Bree, H. Power doppler assessment of the neovascularization during uncomplicated fracture healing of long bones in dogs and cats. *Vet. Radiol. Ultrasound* **2006**, *47*, 301–306.

Jeon, S.; Jang, J.; Lee, G.; Park, S.; Lee, S.K.; Kim, H.; Choi, J. Assessment of neovascularization during bone healing using contrast-enhanced ultrasonography in a canine tibial osteotomy model: a preliminary study. *J. Vet. Sci.* **2020**, *21*, e10

Table S4. Presentation of the standardized histopathological assessment system employed for scaling findings in tissue samples [Šantić et al. 2009 [1]].

Score	Description of Findings
	Bone Allografts Incorporation
0	Intact, no sign of cellular activity
1	Resorptive activity
2	New bone formation
3	Fully incorporated in new bone
	Cortex
0	No sign of cellular activity
1	Resorptive activity
2	New bone formation
3	Bridging to surrounding tissue
	Callus
0	Fibrous
1	Cartilaginous
2	Bony
	Bridging of the Bone Defect
0	No bridging of the bone defect
1	Bridging with fibrous tissue
2	Bridging with fibrous and cartilaginous tissue
3	Bone defect closure
	Newly Formed Bone Tissue
0	No newly formed bone tissue
1	Osteoblasts accumulation
2	Woven bone
3	Lamellar bone
	Periosteum
0	Avascular
1	Vascular

[1] References

Šantić, V.; Cvek, S.Z.; Šestan, B.; Bobinac, D.; Tudor, A.; Miletić, D.; Nemec, B. Treatment of tibial bone defect with rotational vascular periosteal graft in rabbits. *Coll. Antropol.* **2009**, *33*, 43–50.

Figure S1. Histological pictures of metatarsal bone in sheep, 90 days after creation of bone defect and implantation of various biomaterials in there.

(a) Group A: lack of cellular activity, bone defect filled by acellular debris (in apposition to pre-existing bone trabecules) (H & E stain, magnification × 40 (bar: 250 μm)).

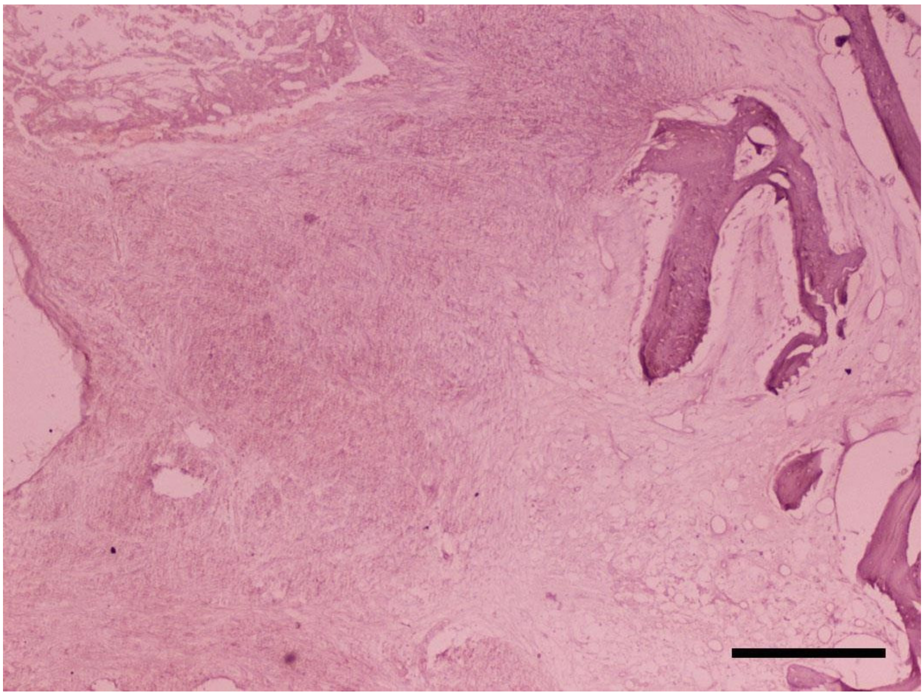

(b) Group B: fibrous callus present in bone defect, bridging achieved by osseocartilaginous trabecules (H & E stain, magnification × 40 (bar: 250 μm)).

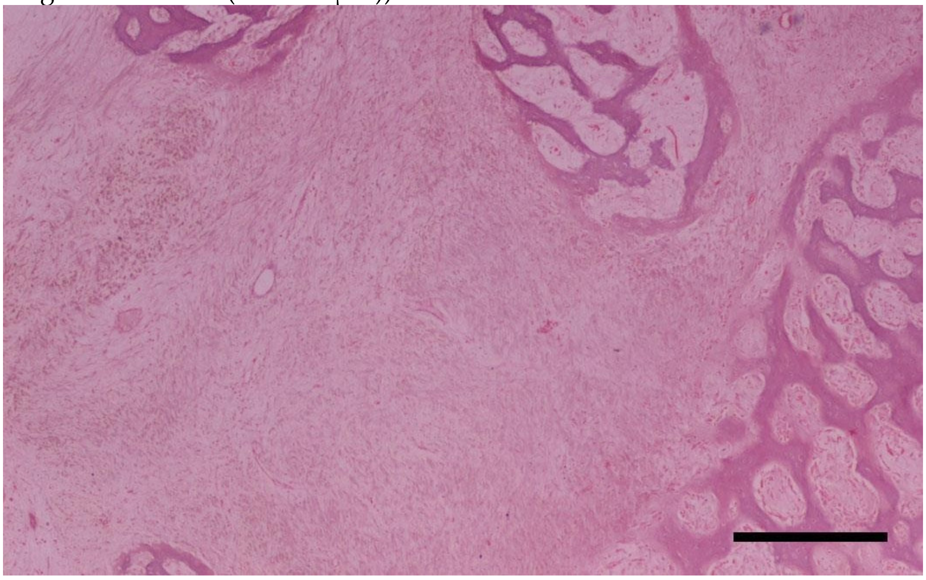

(c) Group C: defect fully closed, formation of new bone filling most of the defect (H & E stain, magnification × 20 (bar: 500 μm)).

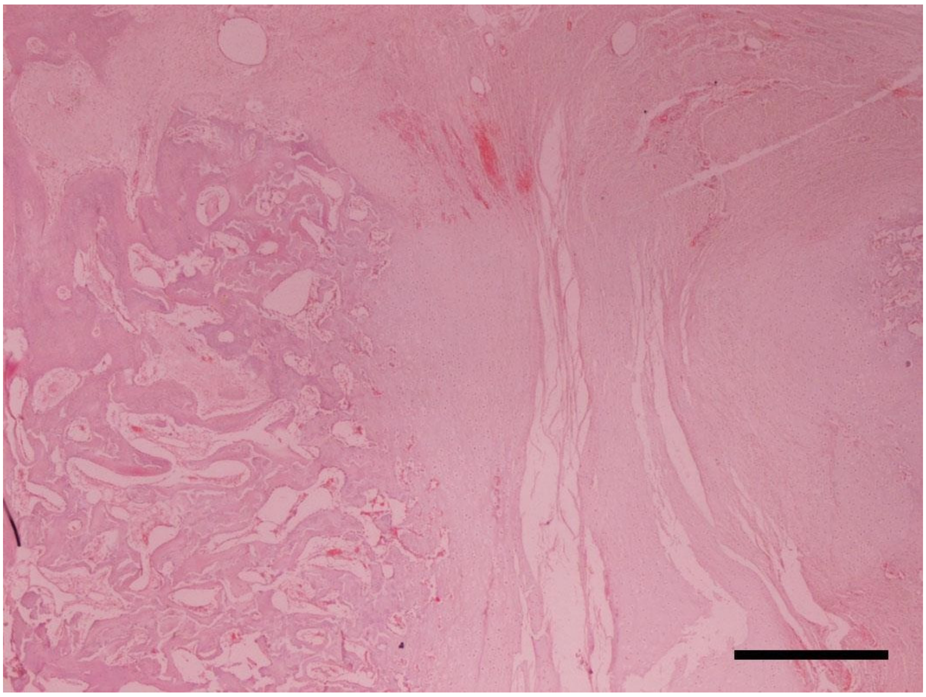

(d) Group D: woven bone filling the bone defect (H & E stain, magnification × 100 (bar: 100 μm)).

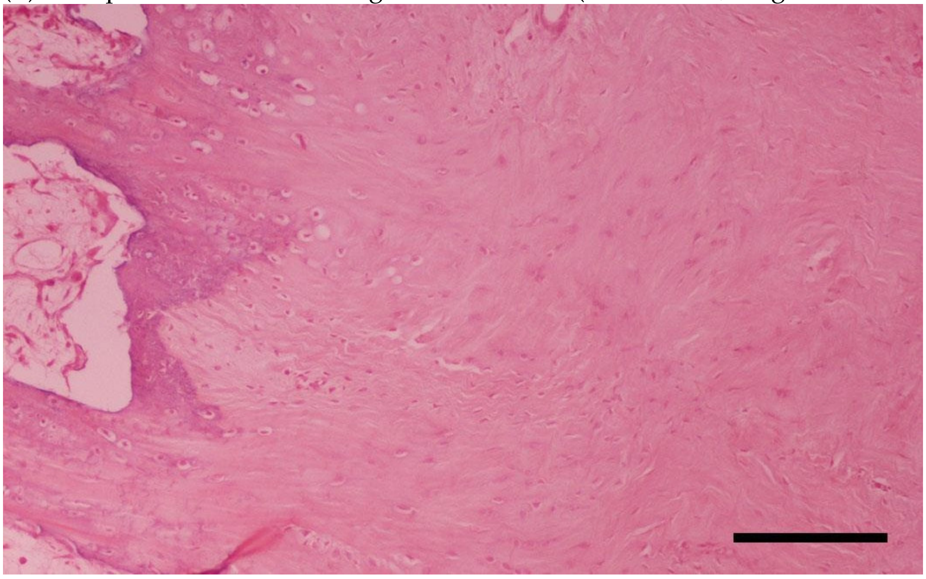

Supplement: Supplementary file 1 [file animals-13-02871-s001.zip › animals-2522763-supplementary.pdf]
